# Editorial: Biofuels and Bioenergy

**DOI:** 10.3389/fpls.2020.621380

**Published:** 2020-11-30

**Authors:** Jin Zhang, Jaime Barros-Rios, Mengzhu Lu

**Affiliations:** ^1^State Key Laboratory of Subtropical Silviculture, School of Forestry and Biotechnology, Zhejiang A&F University, Hangzhou, China; ^2^Biosciences Division, Oak Ridge National Laboratory, Oak Ridge, TN, United States; ^3^Department of Biological Sciences, BioDiscovery Institute, University of North Texas, Denton, TX, United States

**Keywords:** biofuels, bioenergy, biomass, plant cell wall, bioengineering

Plant cell wall biosynthesis is critical for plant growth and development, and a major global carbon sink in the biosphere. Plant secondary cell walls includes three main biological polymers: cellulose, hemicellulose, and lignin (Pauly and Keegstra, 2010). In the past half century, the research on the biosynthesis and metabolism of these three biopolymers has attracted the attention of researchers (Li et al., 2014; Zeng et al., 2014; Kumar et al., 2016; Xie et al., 2018; Polko and Kieber, 2019; Vanholme et al., 2019). In this Research Topic, we aim to gather knowledge about current advances in the field of plant biotechnology for bioenergy production. We have integrated several key components such as technology optimization for biorefineries, genetic modification to improve bioenergy crops, and gene discovery for biofuel and bioenergy production. Together, this knowledge will support the promotion of sustainable biofuels and bioproducts to contribute to the bioeconomy on a global scale.

This Research Topic, Biofuels and Bioenergy, including eight original research articles, four review articles, and one perspective paper, covers the following three topics.

## Technology Optimization for Biofuels and Bioenergy

In the past few decades, cell wall engineering has been a key method to reduce biomass recalcitrance by altering the structure of lignin (Sattler and Funnell-Harris, 2013). Perturbations of genes in the lignin biosynthetic pathway lead to major structural changes, allowing the design of easily controlled biomass structures to produce biofuels and chemicals (Zhang et al., 2019; Fan et al., 2020). In this Research Topic, Kim et al. reported an integrated strategy combining biomass genetic engineering with a pretreatment using a bio-derived deep eutectic solvent (DES). Kim et al. strategically expressed a bacterial hydroxycinnamoyl-CoA hydratase-lyase (HCHL) gene using the lignifying tissue-specific *IRX5* promoter in *Arabidopsis* to reduce the degree of lignin polymerization via incorporation of side-chain-truncated monomers in lignin polymer ends. After pretreatment with the lignin-derived DES at mild conditions, the *IRX5:HCHL-1* transgenic *Arabidopsis* yielded higher levels of fermentable sugars. This approach could support the development of new raw materials for the optimization of the production process in biorefineries.

As one of the main components of plant cell walls, cellulose is the most abundant biopolymer on the planet (Mizrachi et al., 2012). Therefore, understanding the structure of cellulose can provide a basis for improving bioenergy plants by altering the cell wall components. Previous studies using atomic force microscopy (AFM) showed the intricate details of cellulose microfibril arrangement in plant cell walls (Kirby et al., 1996; Ding et al., 2014). However, the image quality is insufficient to resolve the dimension and the cross-sectional shape of cellulose microfibrils. In this Research Topic, Song et al. used the advantages of AFM imaging under aqueous condition and a combined effort of sample preparation, pre-selection of tips with 1 nm radius and systematic adjustment of imaging parameters to optimize image quality, and analyzed the images of primary and secondary cell walls at the sub-nanometer scale.

When compared to the model plant *Arabidopsis*, many species used as bioenergy crops are recalcitrant to both transformation and *in vitro* regeneration (Clifton-Brown et al., 2019). As a fast-growing species, willow (*Salix* spp. L.) has attracted attention due to its potential as a raw material for bioenergy and biofuel production (Nordborg et al., 2018). In this Research Topic, Gomes et al. proposed a reproducible, rapid, and highly efficient *Agrobacterium rhizogenes*-mediated hairy root transformation system for *S. purpurea*. The transformed *Salix* hairy roots can be easily and quickly selected by fluorescent markers. This transformation system provides an effective method of gene function validation for improving bioenergy plants.

In addition to those traditional plants used for bioenergy, more and more plant species are being tested as potential raw materials for bioenergy and biofuel applications. White mustard (*Sinapis alba* L.) is an annual plant of the family Brassicaceae that originates from the Mediterranean region. Mitrović et al. summarized the white mustard seed oil as a promising feedstock for biodiesel production and discussed its fuel properties and performance.

## Genetic Modification to Improve Bioenergy Crops

The ideal bioenergy crop should contain a high proportion of C6-sugars in polysaccharides, and genetic modification of carbohydrate active enzymes can reduce the recalcitrance of the cell wall. In our Research Topic, Brandon and Scheller reviewed progress in the use of a variety of dominant genetic engineering strategies to improve biomass for bioenergy conversion and modulate polysaccharide biosynthesis. Wang et al. expressed a *Hypocrea jecorina* acetyl xylan esterase (*HjAXE*), a member of carbohydrate esterase (CE) family 5, driven by a wood-specific promoter (*PtGT43B* promoter) in hybrid poplar. AX was predicted to deacetylate polymeric xylan in the vicinity of cellulose due to the presence of a cellulose-binding module. Expression of *HjAXE* leads to reduced xylan acetylation and ~30% increased glucose yields in enzymatic saccharification of wood without pretreatment, indicating that the CE5 family could be used as a source of enzymes to reduce biomass recalcitrance in poplar. In addition, Debra-Maceluch et al. evaluated the field performance of several acetylation reduced poplar lines including *HjAXE, AnAXE1* (*Aspergillus niger AXE1*, a CE1 family member) and *RWA* (*REDUCED WALL ACETYLATION*), driven by either a constitutive 35S promoter or a wood-specific promoter. This provides data from early field trials for evaluating genetic modification strategies and assessing the potential pros and cons of using genetic modified crops compared to non-genetic modified commercial crops. In this Research Topic, Mazarei et al. reported that genetic manipulation of a *Panicum virgatum* folylpolyglutamate synthetase gene (*PvFPGS1*) in the one-carbon pathway in switchgrass could lead to improved biofuel production without negatively impacting plant growth and biomass yield.

Moreover, genetic modification of microorganisms used for photo-bioreactors could enhance the efficiency of fermentation. Velmurugan and Incharoensakdi engineered the *Synechococcus elongatus* PCC7942 by knockout ADP-glucose pyrophosphorylase gene *glgC* and insertion of the two pyruvate decarboxylase genes *pdc*-*adh* from two different microorganisms; and increased the ethanol synthesis in the system. This provide another strategy to enhance biofuel products in the fermentation stage.

## Gene Discovery for Biofuel and Bioenergy

Although the transcriptional regulation model of the secondary cell wall has been established in plants in the past 10 years (Zhong et al., 2010; Zhang et al., 2018a), exploring new genes related to the formation of secondary cell wall and their post-transcriptional regulation is still an important issue. In the current Research Topic, Zhang J. et al. summarized recent progress on the transcriptional and post-transcriptional regulatory model of lignin biosynthesis pathway genes in the woody model plant *Populus*. For post-translational protein modification, Sulis and Wang review the phosphorylation, ubiquitination, glycosylation, and S-nitrosylation of transcription factors in monolignols biosynthetic enzymes, and peroxidases in the regulation of lignin biosynthesis and polymerization.

With the rapid development of next-generation sequencing technologies, RNA-Seq data provides a rich resource for gene expression and data mining. Zhang L. et al. analyzed the structural differences of developing xylem in five black poplar cultivars and profiled the transcriptome-wide gene expression. Based on a weighted gene co-expression network analysis, they identified a set of promising candidate regulators for genetic engineering to improve feedstock and enhance biofuel conversion.

Genome-wide association study (GWAS) is an effective strategy using “unstructured” populations to identify significant trait associations with small numbers of candidate genes with high resolution (Porth et al., 2013; Zhang et al., 2018b). In this Research Topic, Chhetri et al. used 869 unrelated *Populus trichocarpa* genotypes from a common garden and tested the association of 25 wood anatomical phenotypic traits and 9 multi-trait combinations with 6.741 million single nucleotide polymorphisms (SNPs). Combining with network-based Lines of Evidence (LOE) method, they identified GWAS hits that are strong gene candidates for experimental validation by integrating data from multiple sources. This study provides insights into the type of genes controlling wood anatomical traits for biotechnological approaches toward optimizing wood traits for biofuel production.

## Concluding Remarks

The 13 articles on this Research Topic only covered a small portion of the current progress in the field of plant biotechnology for bioenergy production. We hope that this collection of research articles contributes with valuable information for researchers and practitioners in the biofuel and bioenergy industry chain. As more researchers work collaboratively on this field, the output will accelerate the development of new bioenergy technologies, and ultimately promote the use of sustainable biofuels in the future bioeconomy.

## Author Contributions

JZ wrote the draft. JB-R and ML made editing and approved the final version for publication. All authors contributed to the article and approved the submitted version.

## Conflict of Interest

The authors declare that the research was conducted in the absence of any commercial or financial relationships that could be construed as a potential conflict of interest.
